# Lung Abscess with a Refractory Bronchopleural Fistula Saved from Potentially Fatal Sepsis by Omentoplasty and Extracorporeal Membrane Oxygenation

**DOI:** 10.1155/2021/9025990

**Published:** 2021-10-21

**Authors:** Jumpei Takamatsu, Jinkoo Kang, Aya Fukuhara, Yuichi Yasue, Sae Kawata

**Affiliations:** Department of Emergency Medicine, Kansai Rosai Hospital, Amagasaki, Japan

## Abstract

Controlling air leaks during thoracic drainage in patients with lung abscesses caused by bronchopleural fistulas is challenging. To reduce the occurrence of air leaks, positive pressure ventilation should be avoided whenever possible. A 69-year-old man presented with a 10-day history of gradually worsening chest pain. He had lost consciousness and was brought to the emergency room. His SpO_2_ was approximately 70%, and his systolic blood pressure was approximately 60 mmHg. Chest radiography and computed tomography revealed findings suggestive of a right pyothorax. Therefore, thoracic drainage was performed. However, the patient's respiratory status did not improve, and his circulatory status could not be maintained. Therefore, extracorporeal membrane oxygenation was introduced after the improvement in circulation by noradrenaline and fluid resuscitation, resulting in adequate oxygenation and ventilation without the use of high-pressure ventilator settings. Subsequently, omentoplasty for a refractory bronchopleural fistula was successfully performed, and the air leak was cured without recurrence of the lung abscess.

## 1. Introduction

Bronchopleural fistula is one of the most common causes of lung abscesses. When a lung abscess is complicated by fatal sepsis, drainage should be performed to control the infection. However, air leakage may occur during drainage when a bronchopleural fistula is present. To reduce air leakage, positive thoracic pressure should be avoided as much as possible. However, in sepsis-associated lung abscesses, respiratory insufficiency necessitates the use of high-pressure ventilator settings to achieve adequate oxygenation and ventilation. In this report, we present the case of a patient in whom a lung abscess and refractory bronchopleural fistula developed into potentially fatal sepsis.

## 2. Case Presentation

**Figure 1:**
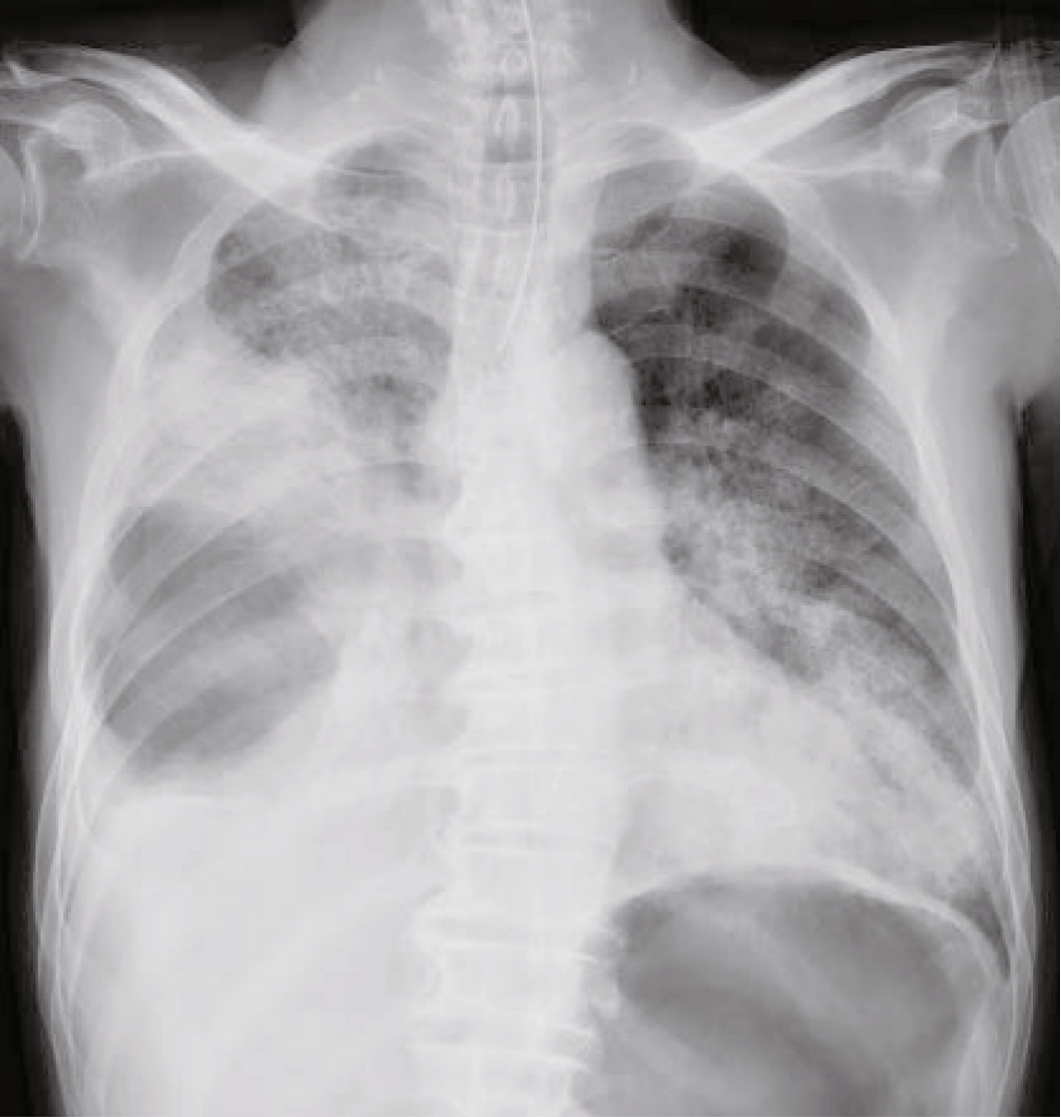
Chest radiograph obtained at admission. The chest radiograph shows pleural effusion and diffuse infiltrative shadows.

**Figure 2:**
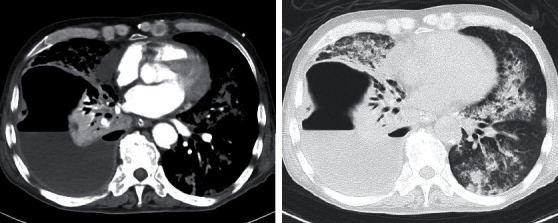
Computed tomography at admission. Computed tomography scans show fluid collection in the right thoracic cavity.

A 69-year-old man was transported to the emergency room after losing consciousness; he had a 10-day history of gradually worsening chest pain. The patient had no history of relevant medical conditions, though it was reported that he smoked cigarettes. After admission to the hospital, the patient's level of consciousness improved to a Glasgow Coma Scale score of E3V4M6; however, his vital signs were unstable (SpO_2_, approximately 70% and systolic blood pressure, approximately 60 mmHg). The patient underwent endotracheal intubation and fluid resuscitation with continuous injection of noradrenaline, which stabilised his circulatory system. Chest radiography and computed tomography (CT) were performed, which led to a diagnosis of right pyothorax (Figures 1 and 2) in addition to septic shock.

**Figure 3:**
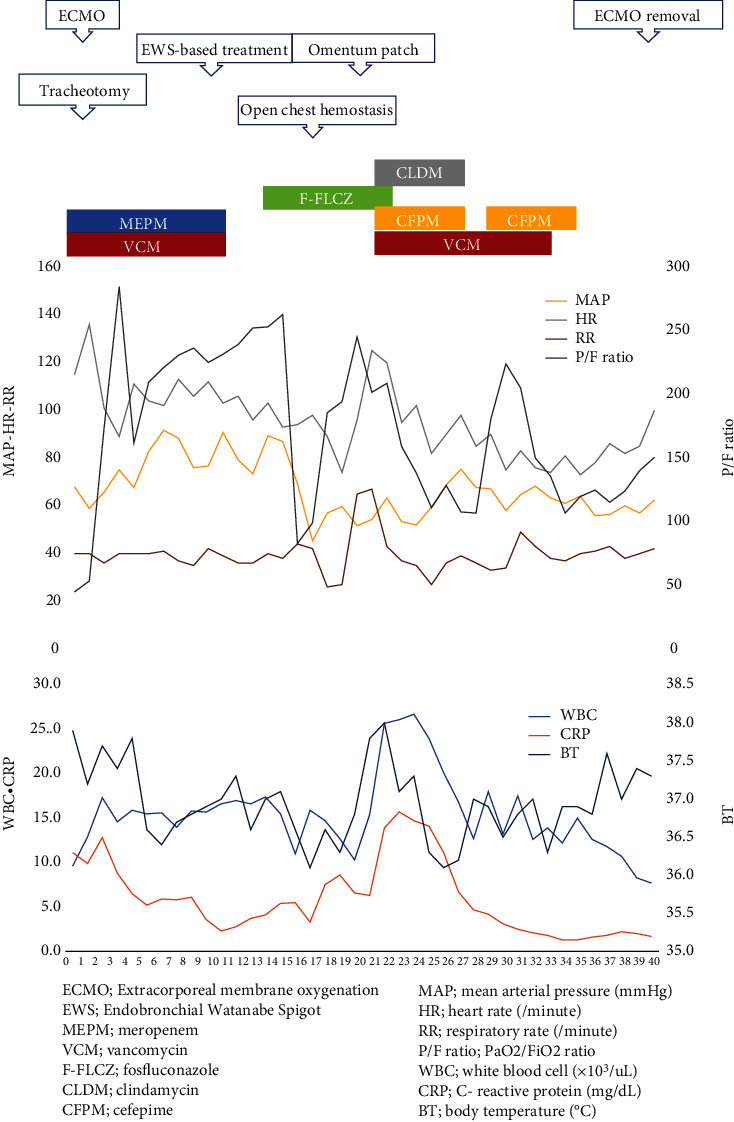
Patient's clinical course. The patient's clinical course during extracorporeal membrane oxygenation (ECMO) is shown. His PaO_2_/FiO_2_ ratio was not favourable throughout ECMO, but his level of consciousness and circulatory dynamics were maintained.

**Figure 4:**
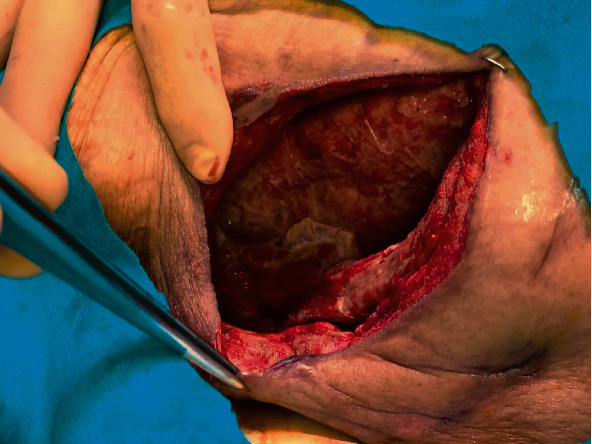
Adherent omental flap. The patient's omental flap is shown to be adherent on day 34 of hospitalisation.

**Figure 5:**
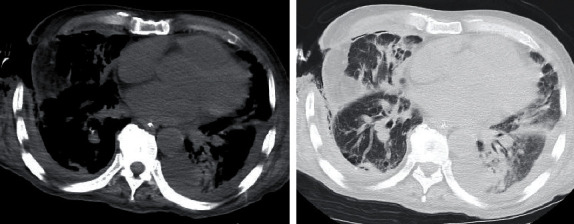
Postoperative computed tomography on day 61 of hospitalisation. Computed tomography scans obtained on day 61 show that the omental flap was viable, and that the abscess had disappeared.

**Figure 6:**
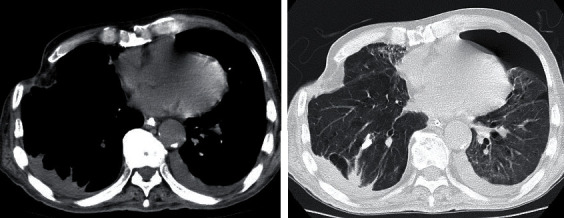
Computed tomography on day 95 of hospitalisation. Improved air flow is observed in the right lung. However, a left pneumothorax is also observed.

The laboratory values obtained on arrival are shown in Table 1. Approximately 400 mL of pus was drained from the right thoracic cavity; however, the patient's respiratory status did not improve. Oxygenation and ventilation could not be maintained, although the patient's circulation was stabilised by fluid resuscitation and noradrenaline. After 12 hours of hospitalisation, extracorporeal membrane oxygenation (ECMO) was introduced via cannulation of the right femoral artery, right femoral vein, and right common jugular vein. The patient was started on veno-venous ECMO as his circulatory system stabilised. We believed that poor oxygenation, in addition to sepsis, was causing worsening of circulation-related problems. Finally, ECMO was performed at 3,000 rpm and a blood flow rate of 2.0 L/min. The patient's clinical course during ECMO is shown in Figure 3. Daily intrathoracic lavage was performed initially, but the air leak from the bronchopleural fistula persisted. On day 10 of hospitalisation, bronchial embolisation with an endobronchial Watanabe Spigot (EWS^®^; HARADA Corporation, Osaka, Japan) was performed without success. On day 15, the patient underwent a thoracotomy for thorough cleaning of the thoracic cavity and abscess. The pleura on the visceral side appeared thickened with unclear borders, suggesting that the abscess was chronic. The mediastinal border was adherent and could not be detached. In addition, an air leak was evident, though the leaking bronchus could not be identified. On day 20, an omentoplasty was performed for the bronchopleural fistula using a pedicled omental flap nourished by the right gastroepiploic artery. The flap was made long enough to reach the thoracic cavity by laparotomy and continued into the thoracic cavity via the anterosternal route by creating a subcutaneous tunnel. The flap was transferred to the bronchopleural fistula and crimped with towel gauze. The chest was then temporarily closed to allow for necessary washing. The air leak was resolved after surgery, and the omental flap became adherent (Figure 4). CT performed on day 61 revealed the disappearance of the abscess and viability of the omental flap (Figure 5). We set the positive end-expiratory pressure (PEEP) of the ventilator to 0 cm H_2_O. On day 40, the patient was weaned from ECMO and treated via ventilatory management. On day 95, the patient was weaned from the ventilator, and CT revealed a left pneumothorax that did not affect his respiratory status (Figure 6). On day 142, the patient was transferred to another hospital to continue rehabilitation for advanced disuse syndrome.

## 3. Discussion

Lung abscesses associated with refractory bronchopleural fistulas require a three-pronged treatment strategy: controlling the infection, treating the respiratory dysfunction, and eliminating the air leak. Antimicrobial therapy is the first choice of treatment for this disease. However, approximately 10% of patients require drainage, and surgery is indicated when drainage fails [1]. The typical operation performed for these patients is partial pneumonectomy or lobectomy. Oxygen is administered to treat respiratory dysfunction; however, mechanical ventilation is required if oxygenation and ventilation cannot be maintained. If the patient's respiratory dysfunction is reversible, ECMO may be performed [2]. Thoracic drainage is required if air leaks develop [3]. However, if the patient's condition worsens to respiratory failure, mechanical ventilation with positive pressure ventilation is required and closure of the fistula is difficult in these patients.

EWS-based treatment is considered for bronchopleural fistulas that do not improve with drainage alone [3]. However, if the fistula does not respond to an EWS through the bronchus, surgical closure of the thoracic cavity should be considered. If occlusion using an EWS is technically difficult, a pneumonectomy that includes the bronchopleural fistula and lung abscess can be performed. However, lung resection is impossible in patients with chronic inflammation and thickened, adherent pleura. In these patients, the bronchial fistula may be closed using a muscle or omental flap [4, 5]. In our patient, an omental flap was used to close the fistula. Omentoplasty is believed to aid in controlling the infection in the bronchopleural fistula due to the anti-inflammatory effects of the omentum [6]. The patient in this case report did not receive PEEP ventilation because ECMO was successfully implemented. Thus, the omental flap was viable, and the bronchopleural fistula was ultimately treated.

The management of respiratory failure due to a refractory bronchopleural fistula with ECMO has been previously reported, and ECMO may be a future management option [7–9]. Although bronchial occlusion with an EWS is effective, it is often ineffective when the bronchi with fistula is difficult to locate [10]. However, if the intrathoracic air leak is covered with an omental flap, precise identification of the location of the leak is not needed. In our patient, ECMO was used to support oxygenation and ventilation, and reduced positive pressure ventilation with a respirator decreased the pressure in the airway, allowing for healing. Daily observation of the patient is required after omentoplasty, as the infection may not respond to the anti-inflammatory properties of the omentum [11]. As our patient was managed with an open chest, the infection status was noted daily.

In our patient, who had a lung abscess caused by a bronchopleural fistula and an air leak that was difficult to control during thoracic drainage, ECMO was used to achieve adequate oxygenation and ventilation without applying high pressure. An omentoplasty over the refractory bronchopleural fistula was successfully engrafted, and the air leak was cured without the recurrence of any lung abscesses.

## Figures and Tables

**Table 1:** Laboratory values at admission.

Item	Result
White blood cells (×10^3^/*μ*l)	9.6
Red blood cells (×10^6^/*μ*l)	3.61
Hematocrit (%)	38.2
Platelet (×103/*μ*l)	335
International normalized ratio of prothrombin time	1.24
D-dimer (*μ*g/ml)	23.02
Total bilirubin (mg/dL)	1.5
Aspartate aminotransferase (U/L)	282
Alanine aminotransferase (U/L)	129
Lactate dehydrogenase (U/L)	450
Sodium (mmol/L)	142
Chloride (mmol/L)	103
Potassium ((mmol/L))	3.7
Blood urea nitrogen (mg/dL)	21.4
Creatinine (mg/dL)	0.84
Albumin (U/L)	1.5
Lactate (mmol/L)	11.8
C-reactive protein (mg/dL)	11.1
PCT (ng/mL)	0.64

## Data Availability

The data used to support the findings of this study are available from the corresponding author upon request.

## References

[1] Mwandumba H. C., Beeching N. J. (2000). Pyogenic lung infections: factors for predicting clinical outcome of lung abscess and thoracic empyema. *Current Opinion in Pulmonary Medicine*.

[2] ELSO (2017). *Extracorporeal Life Support Organization (ELSO) Guidelines for Adult Respiratory Failure; August*.

[3] Morikawa S., Okamura T., Minezawa T. (2016). A simple method of bronchial occlusion with silicone spigots (endobronchial Watanabe Spigot; EWS®) using a curette. *Therapeutic Advances in Respiratory Disease*.

[4] De Weerd L., Endresen P. C., Numan A. T., Weum S. (2019). Intrathoracic breast transposition: a new method in the treatment of bronchopleural fistula and empyema. *Plastic and Reconstructive Surgery. Global Open*.

[5] Okumura Y., Takeda S., Asada H. (2005). Surgical Results for Chronic Empyema Using Omental Pedicled Flap: Long-Term Follow-Up Study. *The Annals of Thoracic Surgery*.

[6] Endoh H., Yamamoto R., Nishizawa N., Satoh Y. (2019). Thoracoscopic surgery using omental flap for bronchopleural fistula. *Surg Case Rep.*.

[7] Grant A. A., Lineen E. B., Klima A., Vianna R., Loebe M., Ghodsizad A. (2020). Refractory traumatic bronchopleural fistula: is extracorporeal membrane oxygenation the new gold standard?. *Journal of Cardiac Surgery*.

[8] Odish M. F., Yang J., Cheng G. (2021). Treatment of bronchopleural and alveolopleural fistulas in acute respiratory distress syndrome with extracorporeal membrane oxygenation, a case series and literature review. *Crit Care Explor.*.

[9] Grotberg J. C., Hyzy R. C., De Cardenas J., Co I. N. (2021). Bronchopleural fistula in the mechanically ventilated patient: a concise review. *Critical Care Medicine*.

[10] Shin K., Hifumi T., Tsugitomi R. (2021). Empyema with fistula successfully treated with a comprehensive approach including bronchial blocker and embolization receiving veno-venous extracorporeal membrane oxygenation. *Acute Med Surg.*.

[11] Yokomise H., Fukuse T., lke O. (1997). Unsuccessful omentopexy in thoracic surgery. *The Thoracic and Cardiovascular Surgeon*.

